# Multiple Dermatofibromas on the Legs

**DOI:** 10.5826/dpc.1004a93

**Published:** 2020-10-26

**Authors:** Gabriel Salerni, Carlos Alonso

**Affiliations:** 1 Dermatology Department, Hospital Provincial del Centenario de Rosario, Universidad Nacional de Rosario, Argentina; 2 Diagnóstico Médico Oroño, Rosario, Argentina

**Keywords:** dermatofibroma, fibrohistiocytoma, polarized light, imaging

## Case Presentation

**Figure 1:**
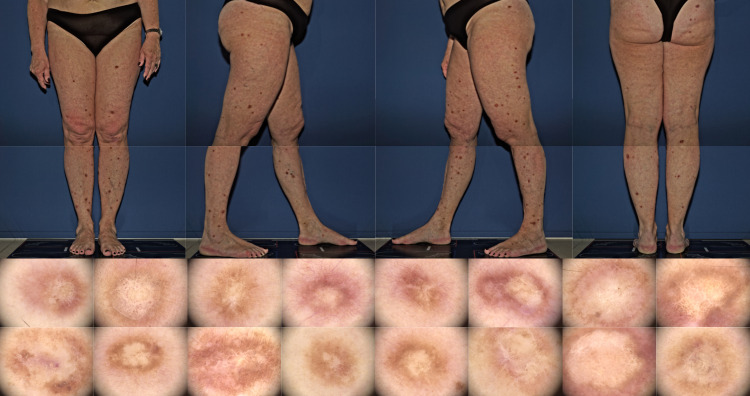
Dermoscopy was consistent with dermatofibroma in all lesions.

A 57-year-old woman presented to consultation complaining of the progressive appearance of skin lesions predominantly distributed in the lower limbs. The spontaneous occurrence of lesions began during adolescence, with continuous appearance of new lesions to the present. The patient was otherwise healthy, and there was no previous history of trauma, autoimmune diseases, immunodeficiency or use of immunosuppressive drugs. Cutaneous examination revealed firm brownish plaques and dome-shaped papules, ranging from 5 mm to 25 mm in diameter, with positive lateral dimple sign. The total number of lesions counted was greater than 180. Dermoscopy was consistent with dermatofibroma in all lesions (Figure 1).

## Teaching Point

While solitary dermatofibromas may be incidental findings, multiple dermatofibromas may be associated with systemic conditions, such as autoimmune diseases, cancer, chromosomal abnormalities, immunodeficiency, metabolic disorders; or previous therapies [1,2]. The presence of many dermatofibromas in a patient without relevant associations, as in this case, is an even less frequent situation.
